# The doing, being, becoming, and belonging (DB3) scale: Development and initial content validity in an Australian context

**DOI:** 10.1111/1440-1630.70074

**Published:** 2026-02-11

**Authors:** Danielle Hitch, Genevieve Pepin

**Affiliations:** ^1^ Occupational Science and Therapy Deakin University Geelong Victoria Australia; ^2^ Occupational Therapy, Western Health St. Albans Victoria Australia

**Keywords:** content validity, occupational being, outcome measurement, pan occupational paradigm, patient‐reported outcome measure

## Abstract

**Introduction:**

Occupational being emerges from interactions between the dimensions of occupation—doing, being, becoming, and belonging. It is a key concept in occupational therapy practice and is also influenced by available resources and opportunities. This study aimed to reflect on the development of a new outcome measure of occupational being—the doing, being, becoming, and belonging (or DB3) scale—and to describe preliminary evidence of its validity and application to occupational therapy, based on content validity testing with occupational therapy academics in Australia and Aotearoa New Zealand and consumers from an Australian health service.

**Methods:**

Applying a descriptive mixed method, this study recruited occupational therapy academics and consumers to complete a single online survey. Content validity was assessed through participants' perceptions of the DB3 scale's applicability, relevance, comprehensiveness, and comprehensibility. Data were analysed using descriptive statistics and Lawshe's content validity ratio, complemented by qualitative data about potential applications to practice.

**Consumer and Community Involvement:**

None.

**Findings:**

Findings indicate that the DB3 scale possesses adequate content validity. All but two of the scale items met the criterion for relevance, and all met the criterion for comprehensibility. The DB3 was perceived by occupational therapy academics as applicable to clinical practice, research, education, and direct service provision with consumers. It was also generally perceived by both groups as comprehensive, although participants recommended some changes which have been incorporated into the final version.

**Conclusion:**

The DB3 scale meets a previously identified need for a tool to translate the core concepts of the Pan Occupational Paradigm (POP) into occupational therapy practice and is the first outcome measure exclusively addressing occupational being. This study underscores its potential utility and applicability across a wide range of settings. Future directions should focus on expanded psychometric evaluation beyond the initial Australian context, exploring cross‐cultural applicability and wider implementation of the DB3 into clinical practice, research, and education.

## PLAIN LANGUAGE SUMMARY

The doing, being, becoming, and belonging (DB3) scale is a new tool. It measures how people feel about their daily tasks, who they are, how they grow and their connections with others. These four areas make up “occupational being.” This idea is the key in occupational therapy. It shows how what we do every day helps build a life that feels good and full of meaning. The DB3 scale asks simple questions. People rate how happy they are with these parts of life. It takes only 5–10 minutes to complete.

We tested the scale with occupational therapy experts in Australia and Aotearoa New Zealand and with everyday people from the community in Australia. We wanted to see if the questions were clear, useful, and easy to answer. The results were positive. Everyone agreed the scale makes sense and works well. It helps show how people join in activities that matter to them. Experts and community members gave helpful feedback. We made a few small changes to create the final version.

Occupational therapists can use the DB3 scale in several ways. They can set goals with clients, track changes over time, and plan treatments that fit each person's needs and wishes. A big strength of the scale is that it comes straight from occupational therapy ideas. It was also built with help from both experts and real people. One limit is that we tested it with a small group. More studies with larger and more varied groups, including people in other countries and cultural contexts, are needed to show how well it works over time. Overall, the DB3 scale is a helpful new way to understand daily life better. It can guide therapists and may shape future therapy practices and rules.

**Key Points for Occupational Therapy**

- The DB3 scale supports occupational therapy practice by evaluating occupational being outcomes.
- Evidence suggests the DB3 scale has acceptable content validity, relevance, and comprehensibility.
- Occupational therapists and consumers consider the DB3 scale applicable to clinical practice, research, education, and working with consumers.

## 1 INTRODUCTION

The complex relationship among occupation, health, and wellbeing is the core business of occupational therapy (Hitch & Pepin, 2021). Many outcome measures developed or adopted in occupational therapy to support assessment and evaluation have arisen from conceptual practice models, related knowledge from other disciplines, or are limited to specific clinical populations (Hitch, 2014; Kielhofner, 2008). Despite extensive outcome measure development in occupational therapy, few have captured the nature of occupational being itself.

Occupational being refers to an identity that emerges from interactions among doing, being, becoming, and belonging—the dimensions of occupation—experienced both individually and collectively (Hitch et al., 2018). This identity is an emergent property of the dimensions of occupation, and occupational being evolves with changes in the inter‐relationships between the dimensions (Hitch & Pepin, 2021). Although often described from an individual perspective (Wagman & Håkansson, 2019), occupational being is also relational and collective, shaped by cultural values, community expectations, and structural conditions (Crawford et al., 2016; Ramugondo & Kronenberg, 2015; Zango Martín et al., 2015). Recognising this social and contextual embeddedness is fundamental to the development and use of measures of occupational being, and to planning interventions that honour both individual experience and shared, collective occupations.

Although the doing, being, becoming, and belonging (DB3) scale has a similar name to the 3B scale developed by Rebeiro Gruhl et al. (2018), these instruments are conceptually distinct. Both scales share common terminology drawn from Wilcock's Occupational Perspective of Health (Wilcock, 1998; Wilcock & Hocking, 2015); however, the 3B scale focusses on occupational balance and participation (2018), whereas the DB3 measures the broader construct of occupational being (Hitch et al., 2018). The title ‘DB3’ was selected for this scale to explicitly represent the four interdependent dimensions of occupation central to this paradigm—doing, being, becoming, and belonging—and to highlight its theoretical lineage, rather than to reference or replicate the 3B scale.

The complexity of occupational being poses significant challenges for outcome measurement, which Rogers and Holm (1994) defined as ‘the functional consequence for the patient of the therapeutic actions implemented by an occupational therapist’ (p. 872). The purposes of outcome measurement are to demonstrate functional change over time and the effectiveness of occupational therapy in enabling these changes (Unsworth, 2000). In broader health outcomes research, Multi‐Attribute Utility Indexes (MAUIs) are widely used to summarise complex constructs such as health‐related quality of life into a single latent variable score based on multi‐attribute utility theory (Baron et al., 2019; Torrance et al., 1982).

Improved occupational being is a critical outcome of occupational therapy for our consumers. For example, international research priority areas for occupational therapy include the effectiveness of interventions, evidence‐based practice, participation in everyday life, healthy ageing, the profession's work with chronic conditions, sustainable community development and population‐based interventions, technology, and professional issues (World Federation of Occupational et al., 2017). Alignment with these research priorities provides a backdrop to the DB3 scale, highlighting the broader momentum towards strengthening occupation‐focussed theory and measurement. The DB3 contributes to this agenda from within its Australian development context, and further research is required to explore whether, and how it resonates in other settings.

This study aimed to describe the development of a new outcome measure of occupational being, the DB3, and present preliminary evidence of its validity and application to occupational therapy. Readers should note that the DB3 was developed and tested within the sociocultural and policy environment of Australia and Aotearoa New Zealand. Interpretation and use of the scale should therefore attend to local histories, service systems, and acknowledge that understandings of doing, being, becoming, and belonging are shaped by place (Doroud et al., 2018; Gallagher et al., 2015; Mathisen & Cele, 2020).

Australia and Aotearoa New Zealand are high income countries with colonial histories. They provide both privately and publicly funded health and disability systems and share a strong policy emphasis on participation in work, education, and community life (Nang & Nang, 2021). Occupational therapy is well established across hospital, community, and rehabilitation services, and occupationally focussed language has been widely incorporated into education and practice. Both countries endorse rights‐based frameworks, including the UN Convention on the Rights of Persons with Disabilities (United Nations, 2006), and have implemented major reforms like the National Disability Insurance Scheme (National Disability Insurance Scheme, 2025) and Enabling Good Lives schemes (Accident Compensation Corporation, 2025) that frame choice, control, and participation as core outcomes. At the same time, the ongoing impacts of colonisation mean that Aboriginal and Torres Strait Islander peoples, Māori, and other minoritised groups continue to experience systemic inequities in health, housing, and occupation (Barbour, 2025), which shape how health, wellbeing, and ‘doing, being, becoming and belonging’ are understood and enacted in practice.

The DB3 was not designed for any specific population but rather as a flexible tool derived from the Pan Occupational Paradigm (POP), which explicitly recognises multiple ways of knowing about occupation (Hitch et al., 2018). As a paradigm, the POP was developed to articulate core dimensions of occupation (doing, being, becoming, belonging, resources, and opportunities) that are relevant to occupational beings across contexts, while recognising that each dimension is expressed and interpreted through specific cultural, social, and historical locations. The DB3 therefore operationalises paradigm level concepts rather than culture‐specific practices and is intended to be further validated and interpreted within, rather than instead of, local contexts.

To orient readers, the remainder of this section first outlines the theoretical foundations and development of the DB3, and then describes the final structure and format of the scale. We then present the methods and findings of the content validity study conducted with occupational therapy academics and consumers.

### 1.1 Development of the DB3 scale

#### 1.1.1 Theoretical foundations

The DB3 scale, developed from the POP, is deeply embedded in occupational therapy principles and responds to requests from therapists for a tangible way to apply the POP in clinical practice (Hitch et al., 2018). The calls for supporting technology from occupational therapists reflect the ongoing development of the paradigm over time, just as its founding framework (Wilcock's Occupational Perspective of Health) has continued to evolve (Wilcock & Hocking, 2015).

The development of the DB3 was shaped by the professional, policy, and sociocultural contexts of Australia, where occupational therapy practice is strongly influenced by universal health‐care coverage (via Medicare, National Disability Insurance Scheme [NDIS] or My Aged Care), its colonial history, geographical diversity, and community‐based rehabilitation models (Brown et al., 2021). These local conditions foreground the importance of equitable access to occupation, the promotion of health and wellbeing through occupation, and the recognition of the structural enablers and barriers that influence occupational being. In these highly multicultural contexts, the item pool drew on international and local literature that included studies with culturally diverse and marginalised groups, and on occupational therapy scholarship on culturally responsive and decolonising practice (Hitch et al., 2014a, 2014b).

Nevertheless, we recognise that further collaborative work with communities whose experiences sit outside dominant occupational therapy traditions is required to fully address potential cultural bias in the tool, and to identify aspects of occupational being that our own professional and cultural locations may have led us to overlook or under‐represent. In subsequent phases of validation, this work will be undertaken collaboratively with communities whose occupational lives, cultural frameworks, and collective practices differ from those represented in this first study. This will include co‐designed exploration of item meaning, testing with diverse cultural groups, including Indigenous and culturally and linguistically diverse communities, and examining whether additional items or adaptations are required. These steps will support the ongoing cultural responsiveness and relevance of the DB3.

As a paradigm, the POP articulates broad assumptions and values rather than specific practice guidelines (Hitch & Pepin, 2021). Paradigms provide the ‘why’ of occupational therapy, whereas conceptual practice models offer the ‘how’, and both can be accompanied by outcomes measures that evaluate their core concept (Kielhofner, 2008). In the case of paradigms, outcomes measures support their verification and validity, drive their ongoing evolution, and enable consistent data collection over time (Kuhn, 2012). The close links between the POP and conceptual practice models were demonstrated by Hitch and Pepin (2021) who mapped how the dimensions of occupation are embedded within multiple occupational therapy models.

The DB3 is founded on the two underlying assumptions of the POP. Firstly, humans are occupational beings whose health and wellbeing are influenced by occupational engagement. This assumption is drawn directly from Wilcock's Occupational Perspective of Health (Wilcock & Hocking, 2015) and articulates the fact that the DB3 was designed to collect information specific to occupational being (and therefore occupational therapy). Secondly, occupational therapy ways of knowing are one perspective among many; other ways of knowing are equally valuable to our understanding of occupation. This assumption reflects a commitment to epistemological equity (Dei, 2008), acknowledging the co‐existence of multiple occupational understandings and the need for conscious action to include marginalised and excluded ways of knowing. In addition, the DB3 mirrors the structure of the POP by incorporating: (1) the occupational dimensions, (2) the occupational being, (3) resources and opportunities, and (4) a health and wellbeing continuum.

The aim of the DB3 is to measure occupational being from the individual (i.e. consumers), group (i.e. therapeutic groups, sporting teams), occupation (e.g. gardening, spiritual practices) and/or role (i.e. parent, student) perspectives (Wilcock and Hocking 2015). The impact of specific occupations, roles, and groups on occupational being can also be explored using four DB3 templates that support flexible application and are freely accessible online (https://osf.io/g52e6/files/osfstorage) (Hitch & Pepin, 2025).

### 1.2 Developer positionality

Both authors identified as female occupational therapy academics residing and practising in Australia. DH was born in Australia, is an English speaker, and identifies as Celtic Australian. GP was born in the French‐speaking part of Canada, speaks both French and English, and identifies as French–Canadian. She acknowledges the influences of both cultures on who she is and what she does. Our understandings of doing, being, becoming, and belonging are shaped by these geographical and cultural locations; by our long‐standing engagement with occupational therapy theory and practice; and by our work in predominantly Western, urban academic and health service environments.

As the originators of the POP and the DB3, we therefore approach this research as ‘insiders’. This proximity offers advantages, including deep conceptual familiarity and sustained engagement with occupational therapy issues, and also risks normalising particular assumptions and overlooking alternative formulations of occupational being. To address these tensions, we purposefully sought feedback from academics and consumers outside our immediate networks, used COnsensus‐based Standards for the selection of health Measurement INstruments (COSMIN) collaboration guidelines (Terwee et al., 2018) to structure the appraisal of content validity, and employed reflexive memoing and collaborative checking of codes and interpretations during analysis to surface and question our assumptions. We return to these positional considerations in the description of our analytic approach and in the strengths and limitations of the study.

### 1.3 Key concepts

*The Dimensions of Occupation*. Four subscales in the DB3 align with the four dimensions of occupation—doing, being, becoming, and belonging (Hitch et al., 2018; Wilcock & Hocking, 2015). The items of these subscales were initially developed from the comprehensive literature review undertaken to develop the POP (Hitch et al., 2014a, 2014b) and were reaffirmed by an updated literature review in 2020. The authors also continue to monitor new research which cites the Occupational Perspective of Health (Wilcock, 1998, 2006; Wilcock & Hocking, 2015) or the POP (Hitch, 2014; Hitch & Pepin, 2021; Hitch et al., 2018) to identify and explore new aspects of these concepts which may emerge over time. The process of both POP and DB3 development has been subjected to repeated and rigorous peer review as part of the first author's PhD studies and subsequent peer review publications and presentations.

The items on the DB3 are abstract in nature and may be open to interpretation. However, consumers are provided with a plain language version of the definitions of these items with prompts developed by Hitch et al. (2014a, 2014b) to support a consistent understanding of what they mean in the context of the DB3 (see Table S1). For example, physical tasks are followed by ‘moving, reaching, and carrying’.

*Occupational Being*. Given that occupational dimensions are continually in flux, occupational being is not a fixed or static entity—it reflects the context, preferences, skills, and needs of the individual, group, or population at that time. These changes over time can be evaluated against the health and wellbeing continuum in conjunction with the final sub‐scale. DB3 scores should be read with attention to relational, cultural, and structural contexts. Low or high scores may reflect collective or community dynamics (e.g. role expectations, shared practices, place‐based belonging), in conjunction with or independent of personal attributes or choices.

*Resources and Opportunities Sub‐scale*. The fifth DB3 sub‐scale is external to the occupational being and describes the more tangible entities which manifest through aspects of the external environment to determine the occupational being's access to occupation. Resources are the available assets (including finances, objects, social capital, and other materials) which enable or disable occupational engagement. Opportunities emerge when elements like networks, change over time, goal‐directed actions, necessity, and chance occurrences contribute to environmental conditions that enable or disable occupational engagement. Occupational engagement depends on having both the resources and opportunities to engage in occupations. This distinction supports clinical reasoning that pairs person‐level strategies with system‐facing action when resources and opportunities are barriers. For example, a person may score low on ‘doing’ for community sport and report low ‘opportunities’ because of transport gaps and fees. Goal setting and intervention should therefore involve both graded or supported re‐engagement and engagement with the contextual barriers to improve outcomes.

The conceptualisation of these sub‐scales as complimentary to each other also reflects the authors' commitment to occupational justice as a means to support occupational beings disadvantaged by factors beyond their direct control (Wilcock & Townsend, 2014). Locating resources and opportunities implies that occupational beings have direct access to, and agency over, them, and this is often not the case. While occupational therapy primarily targets occupational being, the DB3 scale provides an opportunity to also measure advocacy and activism efforts undertaken by the profession to improve resourcing and opportunities.

Access to resources (e.g. materials, finances, social capital) and to opportunities (e.g. time, enabling policies, inclusive networks) shapes who can engage in occupations and how (Hammell, 2020; Pereira et al., 2020). By separating ‘resources’ and ‘opportunities’ from the four dimensions of occupation, the DB3 highlights structural determinants of occupation and supports justice‐oriented reasoning and practice, such as advocacy and service design. Low scores on these items can therefore signal contextual constraints on occupational being rather than limitations within the person or immediate environment.

Making the resources/opportunities items explicit enables occupational therapists to identify and evaluate their impact on structural contributors to occupational outcomes. Occupational therapy addresses both person and systemic level issues by enacting occupational justice at the micro, meso, and macro levels (Bailliard et al., 2020). In practice, this involves individual or group interventions for doing, being, becoming, and belonging, alongside advocacy, service navigation, or environmental adaptation when contextual constraints exist. At the service level, aggregated resources and opportunity data may inform equity audits, pathway redesign, and commissioning decisions by connecting outcome measurement to quality improvement and population health goals. Evaluating the sensitivity of the resources/opportunities sub‐scale to the implementation of occupational justice measures, such as concessional fees, transport support and improved access policies, is a priority for implementation studies.

Items related to resource and opportunities are presented at the end of the doing, being, becoming, and belonging sub‐scales to highlight their links (and potentially differential relationships) with each occupational dimension. There is no plain language definition for the resources and opportunities sub‐scale, as early users indicated its inclusion in the DB3 was either repetitive or confusing. However, prompts are included with these items to guide interpretations of their meaning—resources (e.g. money, items, tools) and opportunities (e.g. chances, enough time).

*Health and Wellbeing Continuum*. The final component of the DB3 is a continuum from ill‐being to wellbeing (Hitch et al., 2018). The continuum is not related to time—it indicates the occupational being's current health and well‐being status. At the bottom of the continuum are the adverse outcomes of occupational engagement: illness, deprivation, alienation, injustice, and death. At the top are the positive outcomes of occupational engagement: wellbeing, health, happiness, inclusivity, and justice. The arrowhead at the top acknowledges the aims of occupational therapy in moving consumers towards positive outcomes by addressing doing, being, becoming, belonging, resources, and opportunities.

**FIGURE 1:**
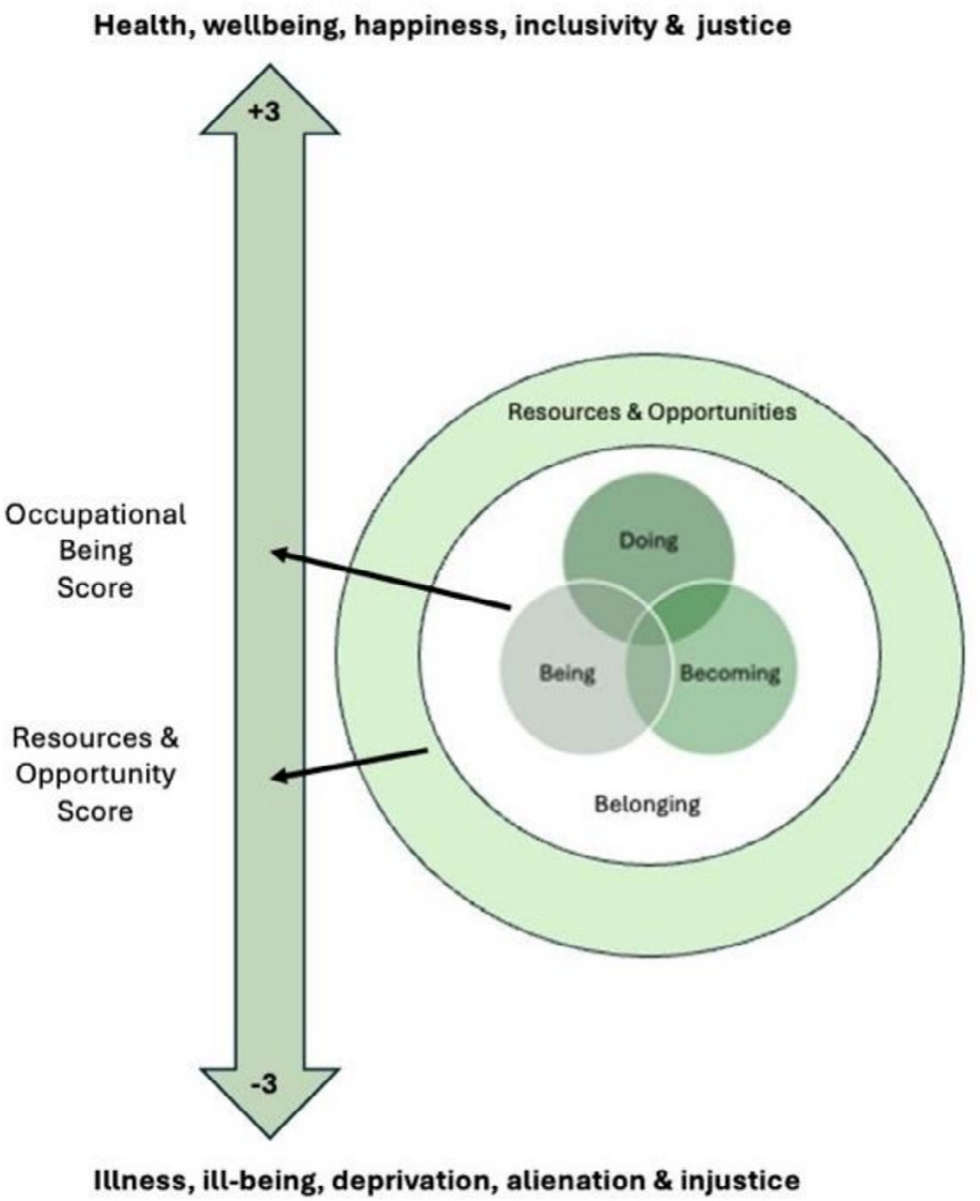
Components of the DB3. DB3, doing, being, becoming, and belonging.

The location of the occupational being on the health and wellbeing continuum can be indicated with the occupational being score and the resources and opportunities score, as shown in Figure 1. An animated version of this figure would show the dimensions pulsing larger and smaller to show how the dimensions are foregrounded at different times and for different occupations. The colours used in this figure are meaningful, as they emphasise the need for sustainable occupations to promote population health.

### 1.4 Structure and format of the DB3 scale

The DB3 scale is a Patient‐Rated Outcome Measure (PROM) (Krogsgaard et al., 2021) which measures the concept of occupational being. It is primarily intended for use as a quantitative outcome measure with individual consumers in clinical and rehabilitation settings but can also be applied to groups, roles, or specific occupations using the DB3 templates described below. The DB3 scale has been validated for consumers over 18 years who can comprehend the items and respond independently. All items are written at Grade 6/Year 7 readability level on the Flesch–Kincaid scale (Kincaid et al., 1975), and it takes approximately 5 minutes to complete.

The DB3 scale contains 42 items across the five sub‐scales (doing, being, becoming, belonging, resources, and opportunities). Respondents rate their satisfaction regarding each item on a 7‐point Likert scale (−3 = *Extremely Dissatisfied*, 0 = *Neutral*, +3 = *Extremely Satisfied*). While a retrospective timeframe of 2 weeks is recommended to complete the scale, the timeframe can be adapted to fit the service context (for example, weekly in acute settings or monthly/quarterly in community and aged care), provided the same timeframe is used consistently within episodes of care. Clinicians should select a period that aligns with expected rates of change and local review cycles and record the chosen timeframe in clinical documentation. Sub‐scale scores are derived from the mean of all item scores, and the Occupational Being score comprises the mean of the doing, being, becoming, and belonging sub‐scale scores. The resources and opportunities sub‐scale score is not combined with any other.

Scores summarise the consumer's satisfaction or dissatisfaction with their occupational being in relation to their doing, being, becoming, belonging, resources, and opportunities. Items which lower scores (i.e. greater dissatisfaction) indicate aspects of occupation which could be targeted during goal setting and intervention, while higher scores identify areas of strength. These DB3 should be completed again after intervention to describe change over time and potentially capture the link between occupational therapy and the consumers' outcomes.

The DB3 can be administered solely as a quantitative scale or as a mixed methods scale. In the mixed methods version, space is provided under each sub‐scale for free text comments by the consumer. This information provides greater context to the data and enhances the information available to occupational therapists. If consumers choose to add comments, the entire scale may take 7–10 minutes to complete.

## 2 METHODS

This study used a descriptive mixed methods design to explore the psychometric properties of the DB3 scale. A single survey was distributed to occupational therapy academics and consumers to capture their perceptions of the content validity of the DB3, with specific reference to its applicability, relevance, comprehensiveness, and comprehensibility as recommended by the (COSMIN) collaboration (Terwee et al., 2018). Content validity refers to the extent to which an outcome measure reflects or evaluates the construct of interest (Mokkink et al., 2010).

Ethics approval for this study was provided by a university (HEAG‐H 78_2020) and a health service (HREC/63606/WH‐2020‐213755). Generative artificial intelligence (AI) tools were used only to support writing clarity and language editing during manuscript preparation. No AI technology was used to generate intellectual content, data, or analysis, and all authors take full responsibility for the integrity and originality of the work.

### 2.1 Recruitment

The inclusion criteria for academics were (1) qualified Occupational Therapist, (2) currently teaching or conducting research in Australian or New Zealand universities providing occupational therapy pre‐registration courses, and (3) able to provide informed consent. The contact details of 204 academic staff were identified on publicly available websites and formed the contact list for this participant group. Academics were therefore recruited via a purposeful strategy which was supplemented by snowball sampling. An invitation to participate (with the plain language statement attached) was emailed directly to these academics, and interested participants were provided with an online link to the survey. Academic participants had 1 month to submit their responses and received two reminder emails in that time.

Consumers were recruited as part of a broader study of occupational being during the initial stages of the COVID‐19 pandemic. The inclusion criteria were (1) people residing in the catchment area of the health service during the timeframe of the study, (2) aged 18 and over, (3) able to understand written English without assistance, (4) able to provide informed consent, and (5) with access to email and internet connection to enable regular response to surveys. Consumer participants may or may not have had prior experience accessing occupational therapy services, allowing us to explore how understandable and relevant the DB3 items were for a range of users.

The study was advertised with an email and attached plain language statement via the volunteer mailing list of a health service and through the professional networks of the research team. It is not possible to determine how many consumers were invited to participate, because of the inclusion of people from a wide geographical area and the purposive nature of the sampling strategy. This approach sought to ensure diversity in age, gender, and occupational experience, consistent with best practice for evaluating content validity (Mokkink et al., 2010; Terwee et al., 2018).

Interested participants registered for the study via an online link, and they completed the DB3 content validity survey. The online registration form included multiple‐choice questions that check participant understanding before they provided their consent, in recognition of the challenges to ensuring informed consent when conducting online surveys (Barrera et al., 2016). Consumers went on to complete the DB3 once a month for the following 6 months, and the findings of the broader study are reported elsewhere. All consumers had 2 weeks to submit their responses, and no reminder emails were distributed.

### 2.2 Data collection

The occupational therapy academic survey began with demographic information about gender, age group, location, and years of experience as an academic. The 42 items of the DB3 scale were then presented and rated according to relevance to its associated sub‐scale (is relevant/is not relevant / unsure of the relevance). Participants were able to identify that additional items they believed should be included in each sub‐scale. Questions regarding the effectiveness and efficiency of the DB3 in supporting occupational therapy practice, education, research, and consumers followed and were rated on a seven‐point Likert scale (*extremely ineffective*/*inefficient* = −3, *neither effective*/*efficient nor ineffective*/*inefficient* = 0, *extremely effective*/*efficient* = 3). Finally, the participants were asked how likely they were to recommend the DB3 to clinicians, educators, researchers, and consumers, and to describe situations where they might apply it to practice. The survey took approximately 10 minutes to complete. Data were collected in anonymous form, thereby protecting participant privacy and confidentiality.

The consumer survey began with demographic information about gender, age group, country of birth, primary language, and whether they had a disability or chronic illness. Their survey included the same questions regarding item relevance and opportunity to identify additional items for each sub‐scale. Data were collected in re‐identifiable form to enable future contact for the purposes of the broader study, but identifiers were removed prior to analysis of the data presented here.

Differences in demographic variables collected for academic and consumer participants reflected both ethical considerations and study intent. Academics were recruited in their professional capacity to evaluate the conceptual and psychometric properties of the DB3, rather than to explore the influence of their personal characteristics on those evaluations. In contrast, the consumer cohort was recruited to assess content validity from a lived experience perspective, making language and disability essential contextual variables for interpreting comprehension and relevance of items. This distinction aligns with accepted practices for establishing instrument content validity, where data collected reflect the participant group's evaluative role (Mokkink et al., 2010; Terwee et al., 2018).

All online surveys were located on the Qualtrics platform (Qualtrics, 2024), and submission of responses was considered implied consent. The participants completed the scale at a single timepoint and reflected on their experiences over the previous 2 weeks.

### 2.3 Data analysis

Quantitative data were analysed using the SPSS package (IBM Corp, 2021) and descriptive statistics such as means and frequencies. Lawshe's (1975) method of quantifying content validity asks a panel of experts to rate items in one of three categories, which in this study were relevant, not relevant, or unsure. The resulting ratio represents a linear transformation of agreement proportions and is calculated as *n*_e_ − (*N*/2)/*N*/2, where *n*_e_ is the number of respondents rating the item as relevant and *N* is the total number of respondents. Ayre and Scally (2014) published updated critical Lawshe ratio values for the number of experts required to agree an item is relevant up to a panel size of 40. The critical values for 40 respondents were applied to all occupational therapy academic responses, as the small number of additional responses (up to *n* = 44) would only have resulted in a lower critical value. The critical values for 17 responses were applied to community member responses to reflect the size of that sample.

Qualitative data regarding comprehensiveness and usability were subjected to content analysis (Vaismoradi et al., 2013) given the relative brevity of the responses and comments. The prevalence of comments was reviewed; they were grouped into similar responses and categorised under themes. All codes were initially developed by DH and then reviewed by GP for consistency and validation. Both quantitative and qualitative data were collected for many of the key variables in this study, and these findings were compared and contrasted in the final stages of analysis.

Rigour and trustworthiness were supported through iterative discussion to confirm category boundaries and reach consensus (Henderson & Rheault, 2004). Reflexive memoing was used throughout the analysis to capture evolving interpretations and potential assumptions, including how our roles as DB3 developers and occupational therapy academics might shape our judgements about item relevance, conceptual boundaries, and perceived gaps. We used this reflexive record to challenge potential blind spots and ensure the final themes reflected both convergence and divergence in interpretation.

## 3 RESULTS

A total of 66 academics (response rate 32%) and 19 consumers submitted the surveys. However, only 44 and 17, respectively, completed all questions; valid response numbers are reported below (see Table 1). Academics from all but one Australian state and territory responded, along with four colleagues from New Zealand. All consumers were recruited from locations in the western suburbs of Melbourne.

**TABLE 1:** Sample characteristics.

Occupational therapy academics			Community members		
		*n*, %			*n*, %
Gender	*Women*	57, 86.7	Gender	*Women*	18, 94.7
	*Men*	9, 13.6		*Men*	0, 0.0
				*Non binary*	1, 5.3
Age group	*18–34*	10, 15.1	Age group	*18–34*	6, 31.6
	*35–44*	11, 16.7		*35–44*	7, 36.8
	*45–54*	33, 50.0		*45–54*	0, 0.00
	*55–64*	11, 16.7		*55–64*	3, 15.8
	*65+*	1, 1.5		*65+*	3, 15.8
Location	*Australia*	96, 93.9	Country of birth	*Australia*	18, 94.7
	*New Zealand*	4, 6.1		*Other*	1, 5.3
Years exp.	*0–9*	24, 36.4	Language	*English*	19, 100.0
	*10–19*	18, 27.3		*Other*	0, 0.0
	*20–29*	14, 21.2			
	*30+*	10, 15.2			
			Disability	*Yes*	5, 26.3
				*No*	14, 73.7

Abbreviation: exp. = experience.

### 3.1 Occupational therapy academics

All but two of the items on the DB3 met the criterion for relevance as measured by Lawshe's ratio (Table 2). These items were related to having the resources and opportunities to ‘do’, and all the resources and opportunities scale items had lower ratios.

**TABLE 2:** Occupational therapy academic construct validity ratings for relevance.

Item	Relevant, *n* (%)	Not relevant, *n* (%)	Unsure, *n* (%)	Lawshe's ratio
Doing sub‐scale				
Doing things that have personal meaning or value for you (*n* = 43)	42, 97.7​	0, 0.0	1, 2.3	0.954
Doing things that have a purpose or meet a goal (*n* = 43)	42, 97.7​	0, 0.0	1, 2.3	0.954
Doing physical tasks (e.g. moving, reaching, carrying) (*n* = 43)​	36, 83.7	1, 2.3	6, 14.0	0.674
Doing mental tasks (e.g. thinking, remembering) (*n* = 43)	37, 86.0	0, 0.0	6, 14.0	0.721
Doing social tasks (e.g. conversation, eating a meal together) (*n* = 43)	39, 90.7	0, 0.0	4, 9.3	0.814
Doing things that meet your own personal needs (*n* = 43)	42, 97.7	0, 0.0	1, 2.3	0.954
Doing things that meet the needs of other people (e.g. family, friends, co‐workers) (*n* = 42)	41, 97.6	0, 0.0	1, 2.4	0.952
Doing things that help you feel healthy and well (*n* = 44)	42, 95.5	1, 2.3	1, 2.3	0.909
Doing organised things (e.g. planned, specific times) (*n* = 43)	39, 90.7	0, 0.0	4, 9.3	0.814
Doing spontaneous things (e.g. unplanned, spur of the moment) (*n* = 43)	41, 95.4	0, 0.0	2, 4.6	0.907
The resources you have to ‘do’ things (e.g. money, items, tools) (*n* = 43)	24, 55.8	5, 11.63	14, 32.6	0.116 **
The opportunities you have to do things (e.g. chances, enough time) (*n* = 43)	26, 60.5	3, 7.0	14, 32.6	0.209 **
Being sub‐scale				
Having time to reflect and contemplate (*n* = 42)	34, 81.0	2, 4.8	6, 14.3	0.619
Your personal sense of self as a human being (*n* = 42)	38, 90.5	0, 0.00	4, 9.5	0.810
Meeting your personal needs (*n* = 42)	34, 81.0	3, 7.1	5, 11.9	0.619
Meeting your spiritual needs (*n* = 42)	35, 83.3	3, 7.1	4, 9.5	0.667
Meeting your cultural needs (*n* = 42)	36, 85.7	3, 7.1	3, 7.1	0.711
Meeting your creative needs (e.g. play, problem solving, art) (*n* = 42)	35, 83.3	3, 7.1	4, 9.5	0.667
Your life roles (e.g. child, partner, student, worker, carer) (*n* = 42)	39, 92.9	1, 2.4	2, 4.8	0.857
Making choices about what you do (*n* = 42)	34, 81.0	1, 2.4	7, 16.7	0.619
Making use of your existing skills (*n* = 42)	31, 73.8	4, 9.5	7, 16.7	0.476
The resources you have for ‘being’ (e.g. money, items, tools (*n* = 42)	29, 69.1	6, 14.9	7, 16.7	0.391
The opportunities you have to ‘be’ (e.g. chances, enough time) (*n* = 42)	33, 78.6	3, 7.1	6, 14.3	0.571
Becoming sub‐scale				
Learning new skills and talents (*n* = 40)	38, 95.0	0, 0.00	2, 5.00	0.900
Your potential to grow and develop as a person (*n* = 41)	38, 92.7	1, 2.4	2, 4.9	0.854
Your potential to grow and develop with other people (*n* = 41)	33, 80.5	2, 4.9	6, 14.6	0.610
Your aspirations or hopes for the future (*n* = 41)	39, 95.1	0, 0.00	2, 4.9	0.902
Changes you want to make for yourself (*n* = 41)	40, 97.6	0, 0.00	1, 2.4	0.951
Changes you want to make with others (*n* = 40)	35, 87.5	1, 2.5	4, 10.0	0.750
Your ability to adapt to change (*n* = 41)	34, 82.9	3, 7.3	4, 9.8	0.659
Maintaining your current skills and talents (*n* = 41)	31, 75.6	6, 14.6	4, 9.8	0.512
The resources you have to ‘become’ (e.g. money, items, tools) (*n* = 40)	32, 80.0	3, 7.5	5, 12.5	0.600
The opportunities you have to ‘become’ (e.g. chances, enough time) (*n* = 41)	35, 83.4	2, 4.9	4, 9.8	0.707
Belonging sub‐scale				
Belonging with partners (i.e. husband/wife, boyfriend/girlfriend) (*n* = 41)	37, 90.2	1, 2.5	3, 7.3	0.805
Belonging with family (i.e. parents, children, other relatives, pets) (*p* = 41)	39, 95.1	0, 0.0	2, 4.9	0.902
Belonging with friends (*p* = 41)	39, 95.1	0, 0.00	2, 4.9	0.902
Belonging with other people in my community (*p* = 41)	39, 95.1	0, 0.00	2, 4.9	0.902
Belonging with people of a similar age to you (i.e. your generation) (*p* = 41)	34, 82.9	0, 0.0	7, 17.1	0.659
Belonging with people of a similar cultural or social background to you (*p* = 41)	36, 87.8	1, 2.5	4, 9.8	0.756
Belonging to the location you call home (i.e. local area, town, state, country) (*p* = 41)	39, 95.1	0, 0.00	2, 4.9	0.902
The resources you have to ‘belong’ (e.g. money, items, tools) (*n* = 41)	29, 70.7	5, 12.2	7, 71.7	0.415
The opportunities you have to ‘belong’ (e.g. chances, enough time) (*n* = 41)	34, 82.9	2, 4.9	5, 12.2	0.659

*Note*: * = items not reaching the benchmark for agreed relevance.

#### 3.1.1 The measurement of occupational being

While the DB3 measures satisfaction, the participants also raised other considerations: ‘*The importance level of different areas will vary‐they are important but how important? Is there opportunity to rank them?’* Several participants also commented on a lack of temporality in the items: ‘*having a sense of your present self as part of a coherent narrative over time’*. One participant described this as ‘*historical belongingness’*, and there was a sense of it encompassing more than the current item about ‘Belonging with people of a similar age to you (i.e. your generation)’; ‘*It is not just the people but the doing with and for … knowing you are doing what people have done in this place before you*.’ The participants noted the items reflected a limited cultural perspective on occupation which may or may not be relevant to people from culturally and linguistically diverse or marginalised communities; ‘*This is skewed towards western individualistic sense of self*.’

The participants also offered more general remarks about the DB3. Some indicated a general lack of certainty around the purpose or scope of the DB3; ‘*it isn't quite clear what the “categories” of doing are intended to be*.’ There were also concerns that consumers would not understand the language used in the scale; ‘*Most people are really going to struggle with this concept*.’ Several comments indicated that the explicit occupational focus of the DB3 could be a barrier to its use by consumers; ‘*OTs know, but not necessarily the public*.’

However, the general comments also demonstrated significant variability in the participants' perspectives on the dimensions of occupation; ‘*the distinction between doing and being and becoming is not quite clear, I think*.’ Many of the specific suggestions for inclusion were already addressed (either explicitly or implicitly) by items in other sub‐scales; ‘*I rejected a lot of the items in the list because they point to doing, not being*.’ For example, the participants suggested the inclusion of individual skills and capacity (being); relaxing, resting and reflection (being); objects and places for doing (belonging); choices and prioritisation (being); and social support and connection (belonging) in the doing sub‐scale.

Qualitative feedback from academic participants is summarised below under themes of conceptual clarity, comprehensiveness, and perceived applicability of the DB3.

#### 3.1.2 Perceived relevance and conceptual clarity of DB3 items

These responses to the scale's structure highlight the close interconnectedness between the dimensions of occupation; ‘*Becoming interrelates to being as well as doing, so the kind of person you want to become as well*.’ As a result of a perceived lack of conceptual clarity, respondents expressed concerns that the measurement properties of the DB3 would be ‘messy’ and believed ‘*the list would not stand up in Rasch analysis because they are quite disparate concepts*.’

Comments about subscale content focussed on whether the DB3 captured the breadth of everyday occupations and acknowledged less positive or obligated forms of engagement. The participants suggested additional domains such as activism, cultural and spiritual occupations, and unhealthy but meaningful activities, and noted that the resources and opportunities items appeared to tap a related but distinct construct. These insights reinforced the need to balance coverage with parsimony while retaining a separate resources and opportunities subscale.

#### 3.1.3 Comprehensiveness of subscale content

Suggestions for additional items were made for doing (*n* = 24, 45.3%), being (13, 26.0%), becoming (12, 25.5%), and belonging (18, 38.3%) subscales. Many recommended additions were related to specific categories of occupation including recreation, productivity, leisure, cultural, creative, spiritual, activism and sustainability. Obligated activities and purposeful but unhealthy occupations were also suggested, in recognition that not all occupational engagement is positive; ‘*There is the usual positive slant‐how will people account for things they mindfully and deliberately do that are not good for their own health, don't benefit other people, respond to deficits rather than needs‐violence, addiction​”*.

Items related to resources and opportunity were listed with their associated occupational dimension, and while respondents recognised their links to each, they also perceived them as distinctive from the rest of the items on the sub‐scales; ‘*the resources and opportunities questions might be ‘relevant’ to each scale, but they do tap a completely different construct*’.

#### 3.1.4 Scale effectiveness and efficiency

**FIGURE 2:**
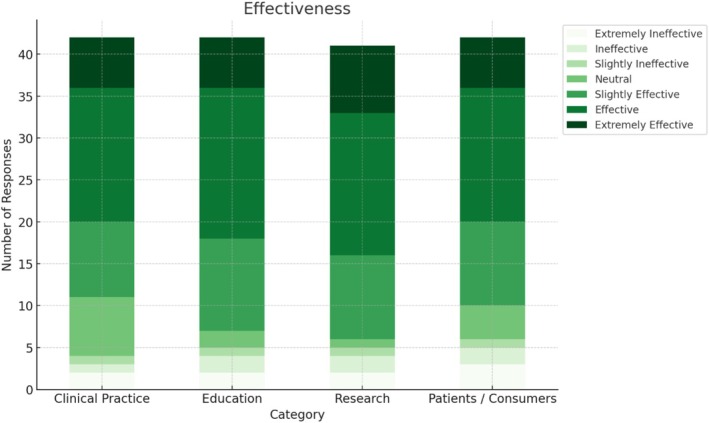
Occupational therapy academics' perceptions of DB3 effectiveness in clinical practice, research, education, and direct work with consumers.

**FIGURE 3:**
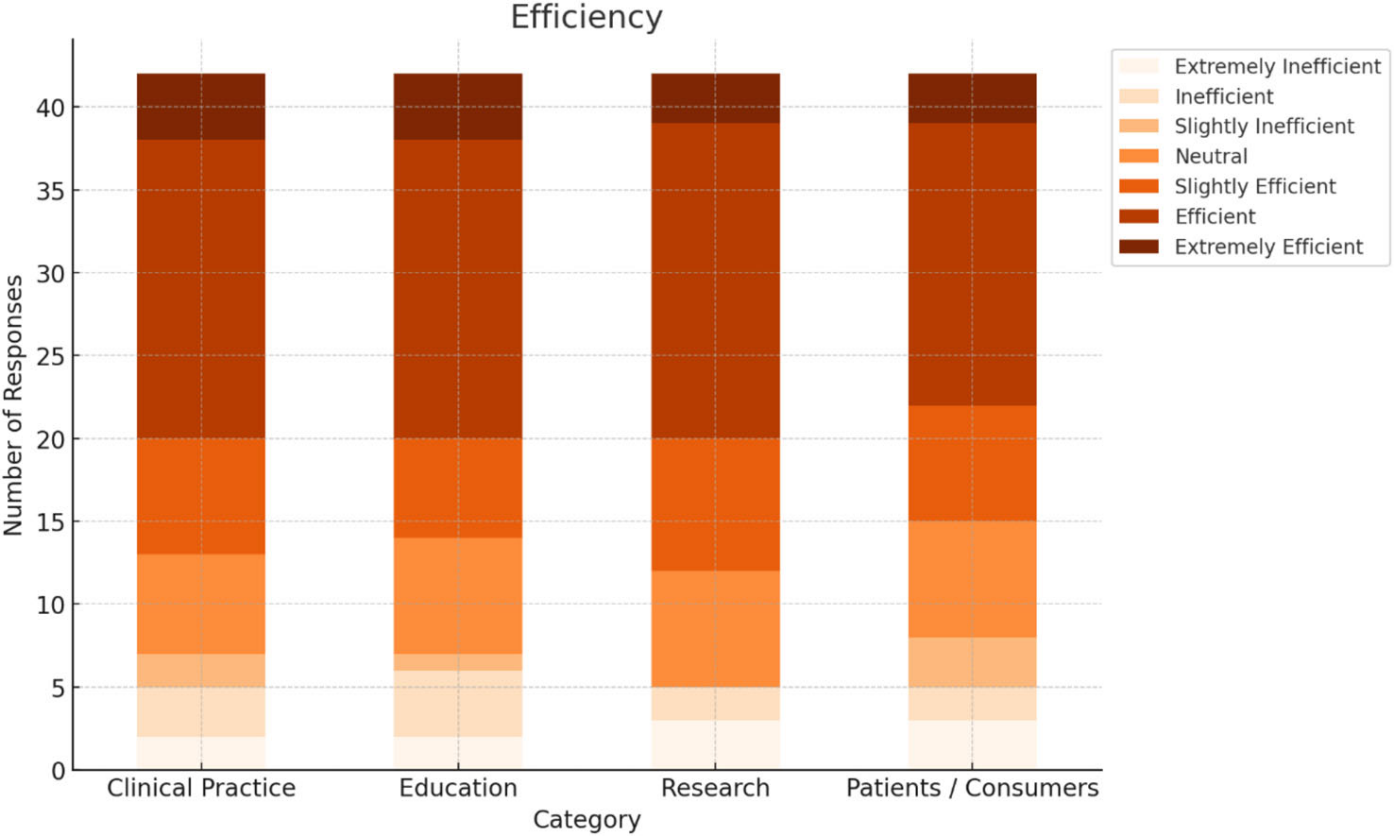
Occupational therapy academics' perceptions of DB3 efficiency in clinical practice, research, education, and direct work with consumers.

Most academics perceived the DB3 as having some degree of effectiveness for clinical practice (*n* = 31, 73.8%), research (*n* = 35, 83.3%), education (*n* = 35, 85.4%), and direct work with consumers (*n* = 32, 76.2%) (Figure 2). A slightly lower proportion perceived the DB3 as being an efficient tool in clinical practice (*n* = 29, 69.0%), research (*n* = 28, 66.7%), education (*n* = 30, 71.4%), and direct work with consumers (*n* = 27, 64.3%) (Figure 3). The following figures summarise the academics' ratings of the DB3's perceived effectiveness and efficiency across four application domains: clinical practice, research, education, and direct work with consumers. Consumer participants contributed perspectives based on their lived experience of occupation and service use rather than from a professional background, and these are reported separately below.

#### 3.1.5 Perceived applicability to practice and education

Most academics were slightly, likely, or extremely likely to recommend the DB3 for use in clinical practice (*n* = 29, 67.4%), education (*n* = 28, 65.1%), research (*n* = 30, 69.8%), and with consumers (*n* = 28, 65.1%). When asked about the potential applications of the DB3, several participants commented they could see the DB3 being applicable to mental health settings, especially because ‘*there are few other tools that really explicitly address ‘being’ and ‘belonging’*. Other populations or settings identified included people with disability, consumers transitioning to new occupational identities after a health event, marginalised groups, and occupational participation patterns in the general community.

Academic participants felt they could use the DB3 as part of initial or more comprehensive assessments, and that it could form part of a suite of outcome measures alongside tools like the Canadian Occupational Performance Measure (COPM). They generally recognised that the DB3 was not designed to be a stand‐alone assessment and would be complemented by other data collection; ‘*If I was using it clinically, I would interview as well‐just the survey response wouldn't be enough for me to work with*.’ The DB3 was perceived as having a potential role in goal setting, strategy development, reflections on recovery, identifying, and readjusting priorities.

Many of the academic participants' recommendations for educational use were related to orienting students to the general principles and concepts of occupational therapy and occupational science. Academic participants also believed the DB3 could support awareness and reflective practice by getting students to engage with their own occupational beings.

#### 3.1.6 Areas for improvement and development

In terms of overall improvements, some participants asked for the DB3 to ‘*drill down to specific examples*’ to avoid it being too theoretical or broad in its scope. ‘*It's a conceptual tool rather than a specific assessment of where anyone is on these scales*.’ They also cautioned against presenting the occupational dimensions as categorical and were concerned consumers would not comprehend the core concepts. ‘*I am not sure they would resonate well with clients and I think the nuances that distinguish between them, would be very hard for clients to understand in a meaningful way*.’ Someone also expressed concern about potential overuse of the terminology. ‘*I think the profession has completely overused these terms and think any further use of them should be very carefully considered‐and not necessarily encouraged. I think their overuse is making them more and more meaningless*’.

The participants recommended future development of the constructs from perspectives located outside dominant Global North and Western traditions (for example, Indigenous knowledges and work emerging from the Global South) (Hayes et al., 2023), along with validation for paediatric or caregiver versions. A ‘not applicable to me’ option was suggested for some items, along allowing for different timeframes for completion in response to the specific needs of consumer groups or practice settings. Several recommendations addressed the wording used in the DB3, which were reviewed and incorporated into the final version of the scale.

### 3.2 Consumers

Qualitative data from consumers were more limited given the smaller sample size, and the number of comments was too small for meaningful content analysis. All the items on the DB3 rated by consumers met the criterion for relevance as measured by Lawshe's ratio (Table 3). The items which attracted lower ratios were related to cultural needs, spiritual need, and belonging with people of a similar cultural or social background. All the DB3 items were rated as understandable by all participants.

**TABLE 3:** Community member construct validity ratings for relevance and understandability.

	Relevance				Understanding			
Item	Relevant, *n* (%)	Not relevant, *n* (%)	Unsure, n (%)	LR	Yes, *n* (%)​	No, *n* (%)	Unsure, *n* (%)	LR
Doing sub‐scale								
Doing things that have personal meaning or value for you (*n* = 19)	19, 100.0	0, 0.0	0, 0.0	1.000	19, 100.0	0, 0.0	0, 0.00	1.000
Doing things that have a purpose or meet a goal (*n* = 19)	19, 100.0	0, 0.0	0, 0.0	1.000	19, 100.0	0, 0.0	0, 0.00	1.000
Doing physical tasks (e.g. moving, reaching, carrying) (*n* = 19)	17, 89.5	1, 5.3	1, 5.3	0.789	19, 100.0	0, 0.0	0, 0.00	1.000
Doing mental tasks (e.g. thinking, remembering) (*n* = 19)	18, 94.7	1, 5.3	0, 0.0	0.895	19, 100.0	0, 0.0	0, 0.00	1.000
Doing social tasks (e.g. conversation, eating a meal together) (*n* = 19)	19, 100.0	0, 0.0	0, 0.0	1.000	19, 100.0	0, 0.0	0, 0.00	1.000
Doing thing that meet your own personal needs (*n* = 19)	18, 94.7	1, 5.3	0, 0.0	0.895	19, 100.0	0, 0.0	0, 0.00	1.000
Doing things that meet the needs of other people (e.g. family, friends, co‐workers) (*n* = 19)	18, 94.7	0, 0.0	1, 5.3	0.895	19, 100.0	0, 0.0	0, 0.00	1.000
Doing things that help you feel healthy and well (*n* = 19)	19, 100.0	0, 0.0	0, 0.0	1.000	19, 100.0	0, 0.0	0, 0.00	1.000
Doing organised things (e.g. planned, specific times) (*n* = 19)	17, 89.5	2, 10.5	0, 0.0	0.789	19, 100.0	0, 0.0	0, 0.00	1.000
Doing spontaneous things (e.g. unplanned, spur of the moment) (*n* = 19)	18, 94.7	1, 5.3	0, 0.0	0.895	19, 100.0	0, 0.0	0, 0.00	1.000
The resources you have to ‘do’ things (e.g. money, items, tools) (*n* = 19)	19, 100.0	0, 0.0	0, 0.0	1.000	19, 100.0	0, 0.0	0, 0.00	1.000
The opportunities you have to do things (e.g. chances, enough time) (*n* = 19)	18, 94.7	1, 5.3	0, 0.0	0.895	19, 100.0	0, 0.0	0, 0.00	1.000
Being sub‐scale								
Having time to reflect and contemplate (*n* = 17)	16, 94.1	1, 5.9	0, 0.0	0.882	17, 100.0	0, 0.0	0, 0.00	1.000
Your personal sense of self as a human being (*n* = 17)	17, 100.0	0, 0.0	0, 0.00	1.000	17, 100.0	0, 0.0	0, 0.00	1.000
Meeting your personal needs (*n* = 17)	17, 100.0	0, 0.0	0, 0.00	1.000	17, 100.0	0, 0.0	0, 0.00	1.000
Meeting your spiritual needs (*n* = 17)	12, 70.6	3, 17.7	2, 11.8	0.412	17, 100.0	0, 0.0	0, 0.00	1.000
Meeting your cultural needs (*n* = 17)	12, 70.6	5, 29.4	0, 0.0	0.412	17, 100.0	0, 0.0	0, 0.00	1.000
Meeting your creative needs (e.g. play, problem solving, art) (*n* = 17)	16, 94.1	1, 5.9	0, 0.0	0.882	17, 100.0	0, 0.0	0, 0.00	1.000
Your life roles (e.g. child, partner, student, worker, carer) (*n* = 17)	16, 94.1	1, 5.9	0, 0.0	0.882	17, 100.0	0, 0.0	0, 0.00	1.000
Making choices about what you do (*n* = 17)	17, 100.0	0, 0.0	0, 0.00	1.000	17, 100.0	0, 0.0	0, 0.00	1.000
Making use of your existing skills (*n* = 17)	17, 100.0	0, 0.0	0, 0.00	1.000	17, 100.0	0, 0.0	0, 0.00	1.000
The resources you have for ‘being’ (e.g. money, items, tools) (*n* = 17)	17, 100.0	0, 0.0	0, 0.00	1.000	17, 100.0	0, 0.0	0, 0.00	1.000
The opportunities you have to ‘be’ (e.g. chances, enough time) (*n* = 17)	16, 94.1	1, 5.9	0, 0.0	0.882	17, 100.0	0, 0.0	0, 0.00	1.000
Becoming sub‐scale								
Learning new skills and talents​ (*n* = 17)	17, 100.0	0, 0.0	0, 0.0	1.000	17, 100.0	0, 0.0	0, 0.00	1.000
Your potential to grow and develop as a person (*n* = 17)	15, 88.2	0, 0.0	2, 11.8	0.765	17, 100.0	0, 0.0	0, 0.00	1.000
Your potential to grow and develop with other people (*n* = 17)	17, 100.0	0, 0.0	0, 0.0	1.000	17, 100.0	0, 0.0	0, 0.00	1.000
Your aspirations or hopes for the future (*n* = 17)	16, 92.4	0, 0.0	1, 5.9	0.882	17, 100.0	0, 0.0	0, 0.00	1.000
Changes you want to make for yourself (*n* = 17)	16, 92.4	1, 5.9	0, 0.0	0.882	17, 100.0	0, 0.0	0, 0.00	1.000
Changes you want to make with others (*n* = 17)	16, 92.4	1, 5.9	0, 0.0	0.882	17, 100.0	0, 0.0	0, 0.00	1.000
Your ability to adapt to change​ (*n* = 17)	15, 88.2	0, 0.0	2, 11.8	0.765	17, 100.0	0, 0.0	0, 0.00	1.000
Maintaining your current skills and talents (*n* = 17)	16, 92.4	1, 5.9	0, 0.0	0.882	17, 100.0	0, 0.0	0, 0.00	1.000
The resources you have to ‘become’ (e.g. money, items, tools) (*n* = 17)	16, 92.4	0, 0.0	1, 5.9	0.882	17, 100.0	0, 0.0	0, 0.00	1.000
The opportunities you have to ‘become’ (e.g. chances, enough time) (*n* = 17)	17, 100.0	0, 0.0	0, 0.0	1.000	17, 100.0	0, 0.0	0, 0.00	1.000
Belonging sub‐scale								
Belonging with partners (i.e. huband/wife, boyfriend/girlfriend) (*n* = 17)	15, 88.2	2, 11.8	0, 0.0	0.765	17, 100.0	0, 0.0	0, 0.00	1.000
Belonging with family (i.e. parents, children, other relatives, pets) (*n* = 17)	17, 100.0	0, 0.0	0, 0.00	1.000	17, 100.0	0, 0.0	0, 0.00	1.000
Belonging with friends (*n* = 17)	17, 100.0	0, 0.0	0, 0.00	1.000	17, 100.0	0, 0.0	0, 0.00	1.000
Belonging with other people in my community (*n* = 17)	16, 92.4	1, 5.9	0, 0.0	0.882	17, 100.0	0, 0.0	0, 0.00	1.000
Belonging with people of a similar age to you (i.e. your generation) (*n* = 17)	17, 100.0	0, 0.0	0, 0.00	1.000	17, 100.0	0, 0.0	0, 0.00	1.000
Belonging with people of a similar cultural or social background to you (*n* = 17)	12, 70.6	2, 11.8	3, 17.7	0.412	17, 100.0	0, 0.0	0, 0.00	1.000
Belonging to the location you call home (i.e. local area, town, state, country) (*n* = 17)	17, 100.0	0, 0.0	0, 0.00	1.000	17, 100.0	0, 0.0	0, 0.00	1.000
The resources you have to ‘belong’ (e.g. money, items, tools) (*n* = 17)	17, 100.0	0, 0.0	0, 0.00	1.000	17, 100.0	0, 0.0	0, 0.00	1.000
The opportunities you have to ‘belong’ (e.g. chances, enough time) (*n* = 17)	17, 100.0	0, 0.0	0, 0.00	1.000	17, 100.0	0, 0.0	0, 0.00	1.000

*Note*: LR = Lawshe's ratio.

Consumers made few suggestions for additional for doing (*n* = 2, 10.5%), being (1, 5.9%), becoming (1, 5.9%), and belonging (1, 5.9%) subscales. Their recommendations included a reference to doing things they are obligated to do, references to specific occupations (such as volunteering), additional examples of aspects of being (including sensual and tactile experiences), and the possibility of online belonging.

## 4 DISCUSSION

This study described the development and initial content validation of the DB3 scale, a novel outcome measure of occupational being grounded in the Pan Occupational Paradigm. By translating the abstract dimensions of doing, being, becoming, and belonging into a brief, clinically feasible tool that complements person‐centred outcome measures, the DB3 offers a practical way to link occupational therapy theory with day‐to‐day decision‐making. The iterative development process, informed by relevant literature and feedback from occupational therapy academics and consumers, supports its face and content validity and provides a foundation for further psychometric testing.

Many academic participants commented on the difficulty of operationalising abstract concepts like doing, being, becoming, and belonging. Categorical representations of occupational therapy theories risk oversimplification and reflect outdated positivist perspectives on occupation and health (Reid et al., 2020). However, the measurement of latent variables using outcome measures can assist in unpacking the dynamic complexity that produces emergent properties (Bandeen‐Roche et al., 1997). The DB3 provides a tangible and accessible entry point to understanding the influence of doing, being, becoming, belonging, resources, and opportunities on occupational engagement, which should be supplemented with the use of additional outcome measures developed from conceptual practice models.

Translating an inherently dynamic construct such as occupational being into 42 items necessarily involves abstraction and runs the risk of reductionism (Backman, 2005). Study participants also described how occupational being is socially and collectively constituted through culture, relationships, and place. The DB3 is not intended to fully describe the richness of occupation; however, it provides a structured entry point for reflection and action on theory, evidence, and practice. Occupational therapists should therefore combine the DB3 with qualitative data collection to retain complexity while benefiting from measurement. The DB3's acknowledgement of marginalised perspectives is grounded in the POP's commitment to epistemological equity (Dei, 2008), which treats diverse ways of knowing about occupation as equally valid. The ‘resources and opportunities’ subscale then helps therapists translate this into practice by highlighting structural barriers to occupational being and prompting occupational justice‐oriented reasoning and action (Bailliard et al., 2020; Wilcock & Townsend, 2014). While the DB3 was informed by international literature, its current form has emerged from occupational therapy practice in Australia and Aotearoa New Zealand (Hitch et al., 2014a, 2014b). This reflects the common pattern in outcome measure development, where tools originate in one cultural context and are subsequently validated in others. We view this study as the first step in a phased validation programme that requires further testing across diverse cultural, linguistic, and service contexts. Such work will allow the DB3 to be empirically examined and culturally interpreted in collaboration with local communities, ensuring both conceptual integrity and contextual responsiveness.

The DB3 is designed to sit alongside person‐centred outcome measures (for instance, the COPM) rather than replace them. While tools like the COPM identify priority occupations and track changes in performance and satisfaction, the DB3 draws attention to how doing, being, becoming, and belonging are experiences simultaneously across time. For instance, an occupational therapist working in hand therapy might use the DB3 at key stages of rehabilitation to explore how surgery, splinting, and exercise are affecting the person's sense of identity, future possibilities, and connection with others. This occupation‐based insight enables the therapist to tailor their person‐centred approach so that physical recovery is aligned with identity reconstruction and participation in meaningful roles, rather than focussing solely on impairment‐level outcomes. When used alongside interviews, narrative approaches and culturally grounded assessments, the DB3 provides a shared conceptual frame for discussing occupational being, while those complementary methods elicit the specific cultural, relational, and place‐based meanings that give the scores their context.

To further interpret these findings, it is useful to consider parallels with MAUIs, which are outcome measures designed to capture latent variables and emergent properties. MAUIs are based on multi attribute utility theory and enable consumers to report how a broad range of influential factors impact upon their health and wellbeing (Torrance et al., 1982). A common example is quality of life measures which, like the DB3, do not provide specific guidance on how to complete assessment and interventions but are commonly employed by occupational therapists (Baron et al., 2019). Some participants reported struggling with the purpose and scope of the DB3, and quality of life measures may provide a useful analogy for occupational therapists and consumers to understand what it can (and cannot) contribute to their work together.

Another key question arising from this study is what occupational knowledge consumers need to benefit from the DB3. Academic participants expressed concern that consumers might not fully understand the constructs of doing, being, becoming, and belonging, yet consumers in this sample reported that the items were clear and relevant. This mismatch echoes wider debates about ‘deficit models’ of science communication, where people are assumed to lack knowledge rather than recognised as bringing their own expertise (Dawson, 2019; Gross, 1994). The plain language design of the DB3 appears to support shared meaning‐making by inviting people to describe how they experience their lives, rather than imposing technical definitions developed within the discipline.

The findings of this study also raise questions about the nature of ‘expertise’ in outcome measurement. Academic participants tended to focus on theoretical nuance, cultural bias, and psychometric rigour, while consumers focussed more on whether the questions resonated with their lives and felt useful in practice. The DB3 is a PROM and therefore centres lived experience expertise, even as academic and clinical experts shape its development and interpretation. Future work should attend carefully to whose perspectives are privileged in decisions about item content, scoring, and implementation, and engage with scholarship and communities beyond dominant Global North contexts to advance epistemic justice in occupational therapy measurement.

### 4.1 Strengths and limitations

This strength of this study includes the diversity of academics participating, close attention to theoretical rigour during scale development and revision, and the use of the COSMIN standards to guide the evaluation of content validity. However, a key limitation of this study is the relatively small number of consumers who participated. Further testing with a wide range of consumers is needed to fully understand what occupational therapists and consumers get from the DB3 when applied in practice. Future studies may also consider including additional demographic variables for academic participants, such as language or disability status, to further explore potential influences on interpretation and to enhance inclusivity.

Other limitations were the small geographical distribution of consumer participants and the risk of bias inherent in purposive sampling. A further limitation is the risk of reductionism inherent in operationalising complex constructs, and future work should explore how the DB3 works with and alongside more qualitative methods of data collection to retain conceptual depth. Implementation studies should examine how DB3 data inform service redesign and community partnerships when collective occupations are central. As outlined in the Developer Positionality section, our insider roles as POP and DB3 developers are another limitation that may have shaped interpretation.

While many other measurement properties need to be established for the DB3, content validity is considered the most important psychometric property to establish initially (Terwee et al., 2018). Validation should also be pursued in contexts where occupational being is experienced primarily as collective or relational and at the group and population levels. As discussed in Sections 1 and 4, this content validity study provides first‐step evidence from an Australian/Aotearoa New Zealand context and does not yet establish the DB3 as universally valid across cultural or service settings.

Both authors are female occupational therapy academics based in Australia, with extensive experience in occupational science, education, and practice development, and both were involved in the development of the POP and the DB3. Our shared disciplinary grounding, long‐standing professional collaboration, and insider status may have shaped our interpretations of ‘doing, being, becoming, and belonging,’ including which elements of occupational being we treated as self‐evident and which were potentially under‐examined. We utilised several trustworthiness strategies to surface and mitigate these influences (Henderson & Rheault, 2004); however, our small and relatively homogeneous research team also limited opportunities for researcher triangulation and peer debriefing from different standpoints. Future studies led by researchers, practitioners, and communities with different cultural and professional locations, including those situated outside Australian and Canadian occupational therapy traditions, are needed to further strengthen analytic rigour, identify concepts we may have overlooked, and extend cross cultural interpretation of the DB3.

## 5 CONCLUSION

The Doing, Being, Becoming and Belonging (DB3) scale makes changes in occupational being visible and trackable, helping therapists link theory with intervention planning and review. In doing so, it strengthens the rationale for occupation‐based, person‐centred practice across diverse contexts and settings. Grounded in the Pan Occupational Paradigm, the DB3 offers a novel approach to measuring occupational being and reflects the theoretical foundations that informed its development. This preliminary study indicates that the DB3 has adequate content validity based on the perceptions of occupational therapy academics and consumers and suggests it may address an important gap in outcome measurement by providing a tool that evaluates the complex and dynamic nature of occupational being. Together, these findings highlight the DB3's potential utility and applicability across a wide range of settings.

Several lines of further research are needed to fully describe the measurement properties and practical contribution of the DB3. Future studies should examine its use in clinical practice, its perceived value for occupational therapists and consumers, and its potential as a service‐wide outcome measure. Additional work is also required to establish reliability, sensitivity, construct validity, and applicability across diverse populations and cultural contexts, using larger and more diverse samples. In occupational therapy education, the DB3 could be evaluated as a tool to deepen understanding of core occupational concepts and support reflective practice. By making personal and systemic influences on occupational being visible for discussion and action, the DB3 can support therapists and services to align intervention with identity, community roles, and context, without relinquishing the complexity that defines occupation.

## AUTHOR CONTRIBUTIONS

**Danielle Hitch**: Conceptualisation, methodology; validation; investigation; analysis; resources; data curation; writing (original draft); writing (review and editing); visualisation; and project administration. **Genevieve Pepin**: Conceptualisation; methodology; validation; analysis; resources; writing (review and editing); supervision; and project administration.

## CONFLICT OF INTEREST STATEMENT

The authors declare that there are no conflicts of interest in relation to this work.

## ETHICS APPROVAL

Ethics approval for this study was obtained from Deakin University Human Ethics Committee (HEAG‐H 78_2020) and Western Health Human Research Committee (HREC/63606/WH‐2020‐213755).

## Supporting information


**Table S1**: Professional and Plain Language Definitions of the Dimensions of Occupation.

## Data Availability

Data are available from the authors upon reasonable request.

## References

[aot70074-bib-0007] Accident Compensation Corporation . (2025). Living my life service: Operational guideline. Accident Compensation Corporation. https://www.acc.co.nz/assets/contracts/lml-service-og.pdf

[aot70074-bib-0008] Ayre, C. , & Scally, A. (2014). Critical values for Lawshe's content validity ratio: Revisiting the original methods of calculation. Measurement and Evaluation in Counseling and Development, 47(1), 79–86. 10.1177/0748175613513808

[aot70074-bib-0009] Backman, C. L. (2005). Outcomes and outcome measures: Measuring what matters is in the eye of the beholder. Canadian Journal of Occupational Therapy, 72(5), 259–261. 10.1177/000841740507200501 16435586

[aot70074-bib-0010] Bailliard, A. L. , Dallman, A. R. , Carroll, A. , Lee, B. D. , & Szendrey, S. (2020). Doing occupational justice: A central dimension of everyday occupational therapy practice. Canadian Journal of Occupational Therapy, 87(2), 144–152. 10.1177/0008417419898930 31964168

[aot70074-bib-0011] Bandeen‐Roche, K. , Miglioretti, D. L. , Zeger, S. L. , & Rathouz, P. J. (1997). Latent variable regression for multiple discrete outcomes. Journal of the American Statistical Association, 92(440), 1375–1386. 10.1080/01621459.1997.10473658

[aot70074-bib-0012] Barbour, V. (2025). The social and political framework of health. Medical Journal of Australia, 222(1), 3–3. 10.5694/mja2.52561 39799461

[aot70074-bib-0013] Baron, H. , Hawrylyshyn, N. , Hunt, S. S. , & McDougall, J. (2019). Understanding quality of life within occupational therapy intervention research: A scoping review. Australian Occupational Therapy Journal, 66(4), 417–427. 10.1111/1440-1630.12570 30746712

[aot70074-bib-0014] Barrera, A. , Dunn, L. , Nichols, A. , Reardon, S. , & Munoz, R. (2016). Getting it ‘right’: Ensuring informed consent for an online clinical trial. Journal of Empirical Research on Health Research Ethics, 11(4), 291–298. 10.1177/1556264616668974 PMC5334448 27630213

[aot70074-bib-0015] Brown, T. , Bourke‐Taylor, H. , & Isbel, S. (Eds.). (2021). Occupational therapy in Australia: Professional and practice issues (2nd ed.). Taylor & Francis.

[aot70074-bib-0016] Crawford, E. , Turpin, M. J. , Nayar, S. , Steel, E. J. , & Durand, J.‐L. (2016). The structural‐personal interaction: Occupational deprivation and asylum seekers in Australia. Journal of Occupational Science, 23(3), 321–338. 10.1080/14427591.2016.1153510

[aot70074-bib-0017] Dawson, E. (2019). Equity, exclusion and everyday science learning: The experiences of minoritised groups. Routledge.

[aot70074-bib-0018] Dei, G. J. S. (2008). Indigenous knowledge studies and the next generation: Pedagogical possibilities for anti‐colonial education. Australian Journal of Indigenous Education, 37(S1), 5–13. 10.1375/S1326011100000326

[aot70074-bib-0019] Doroud, N. , Fossey, E. , & Fortune, T. (2018). Place for being, doing, becoming and belonging: A meta‐synthesis exploring the role of place in mental health recovery. Health & Place, 52, 110–120. 10.1016/j.healthplace.2018.05.008 29885554

[aot70074-bib-0020] Gallagher, M. , Muldoon, O. T. , & Pettigrew, J. (2015). An integrative review of social and occupational factors influencing health and wellbeing. Frontiers in Psychology, 6, 1281. 10.3389/fpsyg.2015.01281 26388800 PMC4554961

[aot70074-bib-0021] Gross, A. (1994). The roles of rhetoric in the public understanding of science. Public Understanding of Science, 3(1), 3–23. 10.1088/0963-6625/3/1/001

[aot70074-bib-0022] Hammell, K. W. (2020). Making choices from the choices we have: The contextual‐embeddedness of occupational choice. Canadian Journal of Occupational Therapy, 87(5), 400–411. 10.1177/0008417420965741 33256473

[aot70074-bib-0023] Hayes, K. , Dos Santos, V. , Costigan, M. , & Morante, D. (2023). Extension, austerity, and emergence: Themes identified from a global scoping review of non‐urban occupational therapy services. Australian Occupational Therapy Journal, 70(1), 142–156. 10.1111/1440-1630.12844 36193547 PMC10092512

[aot70074-bib-0024] Henderson, R. , & Rheault, W. (2004). Appraising and incorporating qualitative research in evidence‐based practice. Journal, Physical Therapy Education, 18, 35–40. 10.1097/00001416-200410000-00005

[aot70074-bib-0054] Hitch, D. (2014). Dynamic and diverse ways of knowing in mental health occupational therapy. Deakin University. http://dro.deakin.edu.au/eserv/DU:30067342/hitch-dynamic-2014A.pdf

[aot70074-bib-0055] Hitch, D. , Pepin, G. , & Stagnitti, K. (2014a). In the footsteps of Wilcock, part one: The evolution of doing, being, becoming and belonging. Occupational Therapy in Health Care, 28(3), 231–246. 24689506 10.3109/07380577.2014.898114

[aot70074-bib-0056] Hitch, D. , Pepin, G. , & Stagnitti, K. (2014b). In the footsteps of Wilcock, part two: The interdependent nature of doing, being, becoming and belonging. Occupational Therapy in Health Care, 28(3), 247–263. 24694178 10.3109/07380577.2014.898115

[aot70074-bib-0057] Hitch, D. , Pepin, G. , & Stagnitti, K. (2018). The pan occupational paradigm: Development and key concepts. Scandinavian Journal of Occupational Therapy, 25(1), 27–34. 10.1080/11038128.2017.1337808 28589783

[aot70074-bib-0058] Hitch, D. , & Pepin, G. (2021). Doing, being, becoming and belonging at the heart of occupational therapy: An analysis of theoretical ways of knowing. Scandinavian Journal of Occupational Therapy, 28(1), 13–25. 10.1080/11038128.2020.1726454 32091297

[aot70074-bib-0059] Hitch, D. , & Pepin, G. (2025). The doing being becoming belonging (DB3) scale version 1.0. 10.17605/OSF.IO/G52E6 41672487

[aot70074-bib-0025] IBM Corp . (2021). IBM SPSS Statistics for Mac (Version 28) [Computer software]. IBM Corp.

[aot70074-bib-0026] Kielhofner, G. (2008). Conceptual foundations of occupational therapy (4th ed.). F.A. Davis Co.

[aot70074-bib-0027] Kincaid, J. , Fishburne, R. , Rogers, R. , & Chissom, B. (1975). *Derivation of new readability formulas (automated readability index, fog count and flesch reading ease formula) for navy enlisted personnel* (Research Branch Report 8–75). Naval Technical Training Command, Research Branch.

[aot70074-bib-0028] Krogsgaard, M. R. , Brodersen, J. , Christensen, K. B. , Siersma, V. , Kreiner, S. , Jensen, J. , Hansen, C. F. , & Comins, J. D. (2021). What is a PROM and why do we need it? Scandinavian Journal of Medicine & Science in Sports, 31(5), 967–971. 10.1111/sms.13892 33249660

[aot70074-bib-0029] Kuhn, T. S. (2012). The structure of scientific revolutions ((4th ed.). ed.). University of Chicago Press.

[aot70074-bib-0030] Lawshe, C. (1975). A quantitative approach to content validity. Personnel Psychology, 28, 563–575. 10.1111/j.1744-6570.1975.tb01393.x

[aot70074-bib-0031] Mathisen, T. , & Cele, S. (2020). “doing belonging“: Young former refugees and their active engagement with Norwegian local communities. Fennia: International Journal of Geography, 198, 39–56. 10.11143/fennia.83695

[aot70074-bib-0032] Mokkink, L. , Terwee, C. , Patrick, D. , Alonso, J. , Stratford, P. , Knol, D. , Bouter, L. , & de Vet, H. (2010). The COSMIN study reached international consensus on taxonomy, terminology, and definitions of measurement properties for health‐related patient‐reported outcomes. Journal of Clinical Epidemiology, 63(7), 737–745. 10.1016/j.jclinepi.2010.02.006 20494804

[aot70074-bib-0033] Nang, T. , & Nang, M. (2021). Comparison of the international health care systems through the consideration of population health and performance indicators in Canada, Australia and New Zealand: A systematic literature review. International Journal of Scientific and Research Publications, 11(2), 199–205. 10.29322/IJSRP.11.02.2021.p11023

[aot70074-bib-0034] National Disability Insurance Scheme . (2025). Understanding the NDIS. https://www.ndis.gov.au/understanding

[aot70074-bib-0035] Pereira, R. B. , Whiteford, G. E. , Hyett, N. , Weekes, G. , Di Tommaso, A. , & Naismith, J. M. (2020). Capabilities, opportunities, resources and environments (CORE): Using the CORE approach for inclusive, occupation‐centred practice. Australian Occupational Therapy Journal, 67(2), 162–171. 10.1111/1440-1630.12642 31957045

[aot70074-bib-0036] Qualtrics . (2024). Qualtrics survey platform [Computer software] . In Qualtrics International Inc. https://www.qualtrics.com

[aot70074-bib-0037] Ramugondo, E. L. , & Kronenberg, F. (2015). Explaining collective occupations from a human relations perspective: Bridging the individual‐collective dichotomy. Journal of Occupational Science, 22(1), 3–16. 10.1080/14427591.2013.781920

[aot70074-bib-0038] Rebeiro Gruhl, K. , Lacarte, S. , Boucher, M. , & Ledrew, L. (2018). Being, belonging and becoming: Development of the 3B scale. Australian Occupational Therapy Journal, 65(5), 354–362. 10.1111/1440-1630.12468 29603255

[aot70074-bib-0039] Reid, H. A. J. , Hocking, C. , & Smythe, L. (2020). The unsustainability of occupational based model diagrams. Scandinavian Journal of Occupational Therapy, 27(7), 474–480. 10.1080/11038128.2018.1544663 30632860

[aot70074-bib-0040] Rogers, J. C. , & Holm, M. B. (1994). Accepting the challenge of outcome research: Examining the effectiveness of occupational therapy practice. American Journal of Occupational Therapy, 48(10), 871–876. 10.5014/ajot.48.10.871 7825701

[aot70074-bib-0041] Terwee, C. B. , Prinsen, C. A. C. , Chiarotto, A. , De Vet, H. C. W. , Westerman, M. J. , Patrick, D. L. , Alonso, J. , Bouter, L. M. , & Mokkink, L. B. (2018). COSMIN standards and criteria for evaluating the content validity of health‐related patient‐reported outcome measures: A Delphi study. Quality of Life Research, 27(5), 1159–1170. 10.1007/s11136-018-1829-0 29550964 PMC5891557

[aot70074-bib-0042] Torrance, G. , Boyle, M. , & Horwood, S. (1982). Application of multi‐attribute utility theory to measure social preferences for health states. Operations Research, 30(6), 1043–1069. 10.1287/opre.30.6.1043 10259643

[aot70074-bib-0043] United Nations . (2006). Convention on the rights of persons with disabilities. United Nations. 10.1515/9783110208856.203 18348362

[aot70074-bib-0044] Unsworth, C. A. (2000). Measuring the outcome of occupational therapy: Tools and resources. Australian Occupational Therapy Journal, 47, 147–158. 10.1046/j.1440-1630.2000.00239.x

[aot70074-bib-0045] Vaismoradi, M. , Turunen, H. , & Bondas, T. (2013). Content analysis and thematic analysis: Implications for conducting a qualitative descriptive study. Nursing & Health Sciences, 15, 398–405. 10.1111/nhs.12048 23480423

[aot70074-bib-0046] Wagman, P. , & Håkansson, C. (2019). Occupational balance from the interpersonal perspective: A scoping review. Journal of Occupational Science, 26(4), 537–545. 10.1080/14427591.2018.1512007

[aot70074-bib-0047] Wilcock, A. (1998). An occupational perspective of health. SLACK Incorporated.

[aot70074-bib-0048] Wilcock, A. (2006). An occupational perspective of health (2nd ed.). SLACK Incorporated.

[aot70074-bib-0049] Wilcock, A. , & Hocking, C. (2015). An occupational perspective of health (3rd ed.). SLACK Incorporated.

[aot70074-bib-0050] Wilcock, A. A. , & Townsend, E. A. (2014). Occupational justice. In B. A. Boyt Schnell , G. Gillen , & M. Scaffa (Eds.), Willard and Spackman's occupational therapy (12th ed.) pp. 541–552. Lippincott Williams & Wilkins.

[aot70074-bib-0051] World Federation of Occupational Therapist , Mackenzie, L. , Coppola, S. , Alvarez, L. , Cibule, L. , Maltsev, S. , Loh, S. Y. , Mlambo, T. , Ikiugu, M. N. , Pihlar, Z. , Sriphetcharawut, S. , Baptiste, S. , & Ledgerd, R. (2017). International occupational therapy research priorities. Occupational Therapy Journal of Research, 37(2), 72–81. 10.1177/1539449216687528 28081694

[aot70074-bib-0052] Zango Martín, I. , Flores Martos, J. A. , Moruno Millares, P. , & Björklund, A. (2015). Occupational therapy culture seen through the multifocal lens of fieldwork in diverse rural areas. Scandinavian Journal of Occupational Therapy, 22, 82–94. 10.3109/11038128.2014.965197 25580632

