# Supplementary material for: The doing, being, becoming, and belonging (DB3) scale: Development and initial content validity in an Australian context
Source: Aust Occup Ther J. 2026 Feb 11;73(1):e70074. doi: 10.1111/1440-1630.70074 (PMC12893835; doi:10.1111/1440-1630.70074)
Supplement: Supplementary file 1 — Table S1: Professional and Plain Language Definitions of the Dimensions of Occupation. [file AOT-73-0-s001.docx]

**Supplementary Materials**

Supplementary Table 1: Professional and Plain Language Definitions of the Dimensions of Occupation

| Dimension | Professional Definition | DB3 Scale Plain Language Definition |
| --- | --- | --- |
| Doing | “The medium through which people engage in occupations and the skills and abilities needed for doing accumulate across time. Doing involves engaging in personally meaningful occupations, but not necessarily purposeful, healthy, or organised. Doing involves being actively engaged, either overtly (i.e., observable, physical) or tacitly (i.e., mental, spiritual). Doing follows broadly similar patterns across the population, and humans can adapt their doing to greater and lesser degrees according to circumstance” (Hitch et al., 2014, p. 241). | People do many things as part of daily life, both alone and with others. These may include what you want to do, have to do and need to do. |
| Being | “The sense of who someone is as an occupational and human being. It encompasses the meanings they invest in life and their unique physical, mental, and social capacities and abilities. Occupation may provide a focus for being, but it also exists independently of it during reflection and self-discovery. Being is expressed through consciousness, creativity, and the roles people assume in life. Ideally, individuals can exercise agency and choice in their expression of being, but this is only sometimes possible or even desirable” (Hitch et al., 2014, p. 241). | This is who you are, including what you enjoy or find meaningful. Being includes your unique physical, mental, and social skills, your life roles and the choices you make about activities. |
| Becoming | “The perpetual process of growth, development, and change that reside within a person throughout their life. It is directed by goals and aspirations, which can arise through choice or necessity, from the individual or from groups. Regular modifications and revisions of goals and aspirations help to maintain momentum in becoming, as does the opportunity to experience new or novel situations and challenges” (Hitch et al., 2014, pp. 241-242). | People change and develop over time. Everyone has hopes or goals to work towards but change beyond your control can happen. The chance to develop new skills is important, as people adjust to change. |
| Belonging | “A sense of connectedness to other people, places, cultures, communities, and times. It is the context within which occupations occur, and a person may experience multiple belongings at the same time. Relationships are essential to belonging, whether they be with a person, place, group, or another factor. A sense of reciprocity, mutuality and sharing characterise both positive and negative belonging relationships, be they positive or negative” (Hitch et al., 2014, p. 242). | Belonging is your sense of connection with other people, places, cultures, communities, and times. All people have multiple relationships with these things and belonging happens when you experience sharing and mutual benefit. |
